# Early exposure to agricultural pesticides and the occurrence of autism spectrum disorder: a systematic review

**DOI:** 10.1590/1984-0462/2023/41/2021360

**Published:** 2022-09-09

**Authors:** Anna Caroline Cristofoli Bertoletti, Kathleen Krüger Peres, Larissa Slongo Faccioli, Marina Camassola Vacci, Isabella Rosa da Mata, Caroline Joana Kuyven, Simone Morelo Dal Bosco

**Affiliations:** a Universidade Federal de Ciências da Saúde de Porto Alegre, Porto Alegre, RS, Brazil.

**Keywords:** Agrochemicals, Pesticides, Autistic disorder, Health services, Maternal exposure, Systematic review, Agrotóxicos, Substâncias químicas agrícolas, Transtorno do espectro autista, Exposição materna, Revisão sistemática

## Abstract

**Objective:**

The aim of this study was to evaluate the influence of early exposure to agricultural pesticides and their relationship with autism spectrum disorder.

**Data source:**

This systematic review was registered at PROSPERO as CRD42020204842. The subject was systematically analyzed on PubMed, Scopus, and Web of Science databases until April 2021. Only studies with humans with early exposure to agricultural pesticides and diagnosis of autism were included. Exclusion criteria were studies on pesticides for domestic or veterinary use and late exposure. There were no language and time restriction. The quality analysis of the studies used the Newcastle-Ottawa Scale.

**Data synthesis:**

Six case-control studies were included; three of them measured the route of exposure by maternal biomarkers and the others by the residence address. The studies had scores between moderate and high in the quality assessment tool. It was found high rates of association between early exposure to agricultural pesticides and autism and detection limit above the quantification for a sample of polychlorinated biphenyls, hexachlorobenzene, and dichlorodiphenyldichloroethylene.

**Conclusions:**

There is evidence concerning the exposure to agricultural pesticides in early life and the development of the autism spectrum disorder; however, more studies are required to better understand their possible association.

## RESUMO

**Objetivo:**

Compreender a influência da exposição precoce a agrotóxicos de uso agrícola e sua relação com o transtorno do espectro autista.

**Fontes de dados:**

Esta revisão sistemática foi registrada no banco de dados PROSPERO sob número CRD42020204842. Por meio das bases de dados PubMed, Scopus e Web of Science, o assunto foi analisado sistematicamente até abril de 2021. Foram incluídos somente estudos com humanos, sendo critérios de inclusão a exposição precoce a agrotóxicos de uso agrícola e o diagnóstico de autismo e critérios de exclusão agrotóxicos de uso doméstico ou veterinário e exposição tardia. Todos os estudos foram analisados sem restrição de linguagem e tempo. A qualidade dos estudos foi avaliada pela NewCastle Ottawa Scale.

**Síntese dos dados:**

No total, seis estudos caso-controle foram incluídos. Três deles mensuraram a exposição pelos biomarcadores maternos e os demais (50%) pelo endereço de moradia. Os trabalhos apresentaram qualidade entre moderada e alta. Encontrou-se alta taxa de associação entre a exposição precoce à agrotóxicos e o autismo e detecção acima do limite de quantificação para amostras de bifenilas policloradas, hexaclorobenzeno e diclorodifenildicloroetileno.

**Conclusões:**

Existem evidências sobre a exposição a agrotóxicos de uso agrícola precocemente e o desenvolvimento do transtorno do espectro autista, porém mais pesquisas são necessárias para melhor compreensão da associação.

## INTRODUCTION

The autism spectrum disorder (ASD) is a neurobehavioral condition with a complex neurological development defined by prejudiced interaction and social communication, restricted and repetitive patterns of behavior, interests, or activities.^1–3^ The diagnostic system that is most commonly used is the Diagnostic and Statistical Manual of Mental Disorders — 5th edition (DMS-5), published in 2013 by the American Psychiatric Association^1^ e o International Classification of Disease — 11th edition (ICD-11), published in 2019 by the World Health Organization (WHO).^4^

Its etiology is not well understood; however, studies suggest that a wide exposure or multiple exposures to a broad class of conditions can compromise the perinatal and neonatal,^5,6^ such as low birth weight,^7,8^ preterm birth,^8^ advanced age of the parents, gestational diabetes, prior fetal loss, hypertension, proteinuria, pre-eclampsia and maternal edema,^9^ multiple births, the Apgar score of <5 min,^5^ maternal obesity^10^ and family history of mental and neurological disorders,^11^ such as more than one child with autism.^12^

Environmental factors have been identified as risk factors for neurodevelopment disorders, including ASD.^13–15^ In contrast to the genetic factors that are irreversible, the environmental factors are modifiable.^16^ Studies show the increase of the association of early exposure to the environmental toxins with the occurrence of autism, such as air pollution in general^17^ and that caused by vehicles^18^and pesticides.^13,19–23^

The pesticides are used to control retention of agricultural plagues,^24^ although, depending on their class, specific substances can impact negatively on human health^25^ acutely or chronically.^26^

This systematic review aimed to comprehend the influence of premature exposure, from the preconception period until the child's first year of life, especially to agricultural pesticides and their possible relation with ASD.

## METHOD

The systematic review protocol was reported according to the Preferred Reporting Items for Systematic Reviews and Meta-Analyses (PRISMA-P) protocol.^27^ The protocol was registered at the international prospective register of systematic reviews (PROSPERO) database under the registration number CRD42020204842.

“Is there an association between premature exposure to agricultural pesticides and the diagnosis of autism spectrum disorder (ASD)?” was the research question of this systematic review, with the following PICOST: Children of both sexes; Premature exposure to agricultural pesticides; Non-exposure to agricultural pesticides; Diagnosis of Autistic Spectrum Disorder; Case-control or cohort studies; At least one measurement.

To identify potentially relevant studies to the present review, a systematic literature search of databases (PubMed, Scopus, and Web of Science) was conducted in July 2020, using MeSH terms and free terms, resulting in the following search: (“autistic disorder” OR “autism spectrum disorder” OR “Asperger syndrome” OR autism OR autistic OR ASD) AND pesticides. All terms were searched in title, abstract, and keyword. No restrictions were applied regarding language or publication date.

Our research included only articles dealing with humans, and the inclusion criteria were as follows: (a) case-control or cohort studies; (b) early exposure to agricultural pesticides; (c) diagnosis of ASD; (d) information on correct exposure by at least one biological relative or maternal biomarkers; (e) published articles complete and with results. The exclusion criteria were as follows: (a) pesticides for domestic or veterinary use; (b) late exposure; (c) use of a risk screening tool or autistic traits; (d) knowledge of high risk for ASD development or non-typical development; (e) presence of concomitant intervention; (f) other neurological disorders; (g) animal model; and (h) meta-analyses, editorials, and narrative reviews.

The articles were screened in two phases. First, duplicates and triplicates articles were removed. In the first phase, two reviewers (A.B and C.C) independently analyzed titles and abstracts in the electronic database and selected articles to identify potentially eligible articles. In the second phase, three reviewers (A.B, C.C, and M.C) independently analyzed and performed the full reading of the articles selected in the first phase, excluding all the articles that did not meet the eligibility criteria. The bibliographic reference of the analyzed articles was also used as a search. At all stages, a third reviewer (I.M) was consulted in the case of any concerns or disagreements among the other investigators, thus resolving all disputes by consensus.

Data extraction was completed independently by four authors in a Microsoft Excel spreadsheet, pre-tested, and developed specifically for this systematic review. The data from the articles were extracted: a) Author, year, and country; b) Study population; c) Measurement of exposure and outcome; and d) Results. Disagreements were resolved by consensus in a team meeting. The extraction was done in a table format, allowing comparisons between studies.

The Newcastle-Ottawa Scale (NOS) Quality Assessment Form for case-control studies was used for assessing the risk of bias. NOS was used because it is a qualitative assessment tool for non-randomized studies that allows assessment in three spheres, namely, the group's selection (with the evaluation of four items/questions), the comparability of the group's (one item), and the verification of exposure or outcome of interest (three items). In each item, it is possible to score either a single star or not, but only with comparison, it is possible to score up to two. The maximum score for each study is 9 stars.^28^ The higher the score, the greater the methodological quality of the study. The tool was applied by two evaluators independently. Disagreements were subsequently discussed with a third reviewer and assessed until a consensus was reached by all the evaluators.

## RESULTS

**Figure 1:**
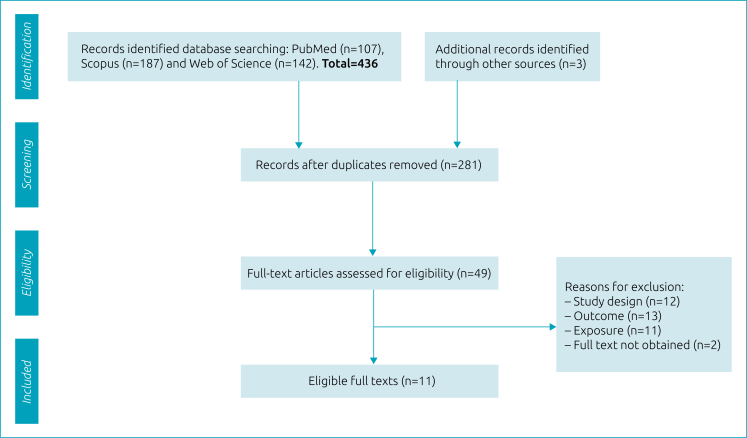
Flow diagram of the systematic review.

A total of 436 potentially relevant studies were identified from the results of the electronic searches (107 from PubMed, 187 from Scopus, and 142 from Web of Science) and another three additional studies were identified from the references of analyzed articles in this systematic review. After removing duplicates and triplicates, 281 records remained for sorting the title and abstract. Of these, 49 complete studies were read in their entirety. After excluding 43 articles that did not meet the inclusion criteria, 6 studies were included.^13,19–23^ A flowchart detailing the process of identification, screening, eligibility, and the inclusion of studies is presented in Figure 1.

Six case-control studies, with data from California^20–23^ and Finland,^13,19^ were selected. Only two of the included articles were derived from cohort studies,^13,19^ but the methodology used was case and control. The sample of the six studies totalized 46,926 individuals: 2,994 cases and 43,932 controls. The sample size ranged from 75^13^ to 545^20^ children with ASD. The way to assess exposure included assessment by maternal biomarkers^13,19,20^ and analysis by home address.^21–23^ The main characteristics of the studies included in this systematic review are described in Tables 1 and 2 by the way of exposure.

**Table 1:** Characteristics of studies that associate exposure to agricultural pesticides with autism by home address — studies cited according to the year of publication.

Author	Study population	Exposure and outcome	Results
Roberts, et al.^21^	Children born between 1996 and 1998, residing in the 19 countries in the Central Valley in California. Cases (n=465) and controls (n=6,975)	Home address of birth and time in pregnancy during which exposure to pesticides reported by data from the California Department of Pesticide Regulation. Children receiving services for autism or with an ASD diagnostic code.	The quantity of organochlorines for the cases suggested OR=6.1 for the ASD. When comparing the fourth quartile versus no exposure, the autism risk coefficient had associations during pregnancy with organophosphates (p=0.042), organochlorines (p=0.025), trifluralin (p=0.046), and bifenthrin (p=0.048). During the embryogenesis of the central nervous system, the results for organochlorines were attenuated with an increase in the distance from home to the application, from 250 m (p=0.001) to 1,000 m (p=0.006).
Shelton, et al.^22^	Children aged 2–5 years, residing in California between 1997 and 2008 with an ASD (n=486), DD (n=168), and typical development (n=316).	Home address for 3 months preconception until born, linked to the use of the California Pesticide Use Report. Previous diagnosis of ASD: ADI-R+ADOS. Typical development and DD: SCQ	Children with ASD were 60% more likely to have organophosphates applied close to the home (aOR=1.60) purchased with GC and increased for exposures in the third trimester (OR=2.0). Cases with DD had aOR = 2.48 of having carbamate pesticides applied close to home during pregnancy. Associations decreased as the size of the beam increased. Exposure to pyrethroid insecticide applications before conception or during the third trimester was at increased risk for ASD and DD (ORs 1.7–2.3).
Von Ehrenstein et al.^23^	Individuals of any age, resident at birth, and diagnosed in California counties. Recruited between 1998 and 2010. Cases (n=296) and controls (n=35,370).	Home address at birth related to data from application reports with evaluation 3 months before pregnancy, during pregnancy, and during the first year of life. Service records are collected through regional centers and categorized by the department, using DSM-IV ed.	Stronger associations between ASD risk and exposure during the development period were for chlorpyrifos (OR=1.15), diazinon (OR = 1.14), and avermectin (OR=1.14). For ASD with ID, exposures resulted in attenuated effect estimates before and during pregnancy, while probability ratios became more pronounced for exposures in the first year of life, for glyphosate (1.60), diazinon (1.45), malathion (1.29), and bifenthrin (1.33). Exposure in the three pre-pregnancy months had weaker associations with ASD. Exposure to any pesticide substance in the first year of life brought an increase in risk for ASD combined with ID by up to 50%.

ASD: autism spectrum disorders; DD: developmental delay; ID: intellectual disability; OR: *Odds Ratio*; aOR: adjusted *Odds Ratio*; GC: control group; GP: general population; DSM-IV: *Diagnostic and Statistical Manual of Mental Disorders*, 4th edition; ADI-R= Autism Diagnostic Interview – Revised; ADOS: Autism Diagnostic Observation Schedule; SCQ: Social Communication Questionnaire.

**Table 2:** Characteristics of studies that associate exposure to agricultural pesticides with autism by maternal biomarkers – studies cited according to the year of publication.

Author	Study population	Exposure and outcome	Results
Cheslack-Postava et al.^13^	Children born in Finland between 1991 and 2000 (n=75 case-control pairs)	Maternal serum biomarkers (collected during the first trimester of pregnancy) analyzed by chromatography. Finnish registry with the diagnosis of *ICD10* F84.0 and subsequent validation with ADI-R.	All samples, except DDT and BDE-47, were measured at levels above the LOQ. Total PCBs at or above the 90th percentile were associated with an OR=1.91 for autism.
Lyall et al.^20^	Children born in three California counties between 2000 and 2003 with ASD (n=545), ID (n=181), and GP (n=418).	Samples of serum and maternal blood cell sediment (collected during 15–19 weeks of gestation) analyzed by chromatography. Previous registration and revised by MADDSP protocol. ASD classified by *DSM-IV* ed. With or without ID (functional cognitive test <70).	In the ASD group, the levels of PCBs and trans-nanochlorines were higher than in the CG. Increased risk of ASD mainly for PCBs 138/158, 153, 170, and 180 (aOR>1.5). The sum of PCB congeners demonstrated a moderately high risk of ASD for the upper quartile (OR=1.39). The trend tests were significant only for PCB 138/158 (p=0.03) and PCB153 (p=0.04). Increased risk of ASD for PCBs was not observed, either crude or adjusted.
Brown et al.^19^	Children born in Finland between 1987 and 2005 (n=778 case-control pairs).	Maternal serum biomarkers (collected between 2 and 4 months of pregnancy) analyzed by chromatography. Hospital record with diagnosis of ICD10 F84.0 and validation with the Autism Diagnostic Interview.	p,p’-DDE was measured in an amount greater than the LOQ in all samples. Among similar PCBs, it was measured above the LOQ between 95% and 100% of the samples. Increased chances of autism in offspring when maternal levels of p,p’-DDE in the 75th percentile (OR=1.32 and p=0.03), male (OR=1.35 and p=0.04), and also individuals with intellectual disabilities (OR=2.21 and p=0.002). No association was observed for PCB 138 or PCB 153 as well as the other total PCBs, congeners, and autism.

ASD: autism spectrum disorders; ID: intellectual disability; OR: *Odds Ratio*; aOR: adjusted *Odds Ratio*; GP: general population; p,p’-DDE: dichlorodiphenyldichloroethylene; PCB: polychlorinated biphenyls; NEQ: neurotoxic equivalent; LOQ: limit of quantification; HCB: hexachlorobenzene; BDE-47: 2,2’4,4’-tetrabromodiphenyl ether; *ICD10* F84.0: International Classification of Disease for Autistic Disorder; DSM-IV: *Diagnostic and Statistical Manual of Mental Disorders*, 4th edition; ADI-R: Autism Diagnostic Interview – Revised; MADDSP: Metropolitan Atlanta Developmental Disabilities Surveillance Program.

The pesticides analyzed by maternal biomarkers were as follows: total polychlorinated biphenyls (PCBs);^13,19^ PCB congener: PCB 29,^20^ PCB 74,^19^ PCB 99,^19,20^ PCB 118, 138, 153, and 156,^13,19,20^ PCB 158,^20^ PCB 170 and 180,^13,19,20^ PCB 183^19^ and PCB 187,^19,20^ PCB 194,^20^ PCB 196/203^20^ and PCB 199,^20^ DDT,^13,20^ dichlorodiphenyldichloroethylene (DDE),^13,19,20^ trans-nonachlor,^20^ hexachlorobenzene (HCB),^13^ and 2,2’4,4’-tetrabromodiphenyl ether (BDE)-47.^13^

Regarding the assessment by place of residence, the following pesticides were found: organochlorines,^21,22^ organophosphates (OPs),^21,22^ bifentrin^21^ and trifluralin,^21^ carbamates,^22^ pyrethroids,^22^ Glyphosate,^23^ chlorpyrifos (CPF),^23^ diazinon,^23^ acephate,^23^ malathion,^23^ permethrin,^23^ bifenthrin,^23^ methyl bromide,^23^ imidacloprid,^23^ avermectin,^23^ and myclobutanil.^23^

The classification score was used according to the meta-analysis by Hu et al.^29^ and was recently replicated in the systematic review by Moura et al.^30^ These studies considered the score 0–3 stars as low methodological quality, 4–6 stars as moderate quality, and 7–9 stars as high quality. According to this classification, the studies included in this systematic review obtained moderate^13,19,21,23^ and high scores,^22,20^ as shown in Table 3.

**Table 3:** Methodological quality of the included studies acoording to the Newcastle-Ottawa Scale.

Study	Selection	Comparability	Exposure	Total
Roberts et al.^21^	**	*	**	5
Shelton et al.^22^	****	*	**	7
Von Ehrenstein et al.^23^	**	**	**	6
Cheslack-Postava et al.^13^	**	**	**	6
Lyall et al.^20^	***	**	***	8
Brown et al.^19^	***	*	**	6

Selection: Maximum 4 points awarded for the case definition, representativeness of the cases, selection of controls, and definition of controls. Comparability: Maximum 2 points awarded for controlling for the pre-specified primary confounding variable (age) and additional confounding variables. Exposure: Maximum 3 points ascertainment of exposure, method of ascertainment for cases and controls, and non-response rate. Total: A maximum of 9 points could be awarded.

NOS-identified selection bias in the case definition found on previous records from other care services, without reassessment of ASD diagnostic tests,^21,23^ the non-explanation of the selection of control cases derived from the cohort,^13^ as well as the lack of similar assessment of individuals were included in the control group.^19,13,20,21,23^

The adjustments made by Cheslack-Postava et al.,^13^ Lyall et al.,^20^ and Von Ehrenstein et al.^23^ included matching factors that were considered extremely important by the authors of this systematic review. The others^19,21,22^ made less specific adjustments, such as socio-demographic indices, ethnicity, and education.

In measuring exposure, three of the articles verified exposure by housing registration^21–23^ that was considered a form of self-report. In this same sphere of evaluation, a lack of information on sampling losses was detected.^13,19^

## DISCUSSION

The results of this systematic review show high rates of association between early exposure to agricultural pesticides and autism, and samples of PCBs, HCB, and DDE had detection limit above the quantification being assessed for maternal biomarkers or home address.

The study by Roberts et al.^21^ demonstrated that the quantity of organochlorines for the cases of ASD was found to be OR=6.1. When comparing the fourth quartile versus no exposure, the risk factor for autism had association with OPs (p=0.042), organochlorines (p=0.025), trifluralin (p=0.046), and bifenthrin (p=0.048) during pregnancy. During the embryogenesis of the central nervous system (CNS) (days 7–49), the results for organochlorines were reduced with an increase in the distance of 250–1,000m (p=0.001 to p=0.006, respectively) from home to the application of the pesticides.

Environmental exposure to certain categories of agricultural pesticides can increase the risk of neural tube defects (NTDs), with effect estimates of less than 1,000m for residential proximity, as demonstrated in the study by Rull et al.,^31^ in which he further suggested the increase in anencephaly associated with organophosphorus pesticides and spina bifida with amides, benzimidazoles, and methyl carbamates. Exposed mothers with combinations of two or more pesticides may be at increased risk of having a baby affected by NTDs.

Corroborating the findings, the systematic review by Muñoz-Quezada et al.^32^ reinforces the hypothesis that agricultural pesticides act negatively on neural and behavioral development in children, when exposed during intrauterine or in the first year of life, as the exposure to OP is associated with neurodevelopment in children. Of the 27 articles analyzed, 26 associated negative effects of pesticides on neurobehavioral development.

The study by Shelton et al.^22^ evaluated the application of agricultural pesticides and OPs, which were the most commonly applied pesticides close to home during pregnancy, which had been identified in the following order: CPF (20.7%), acephate (15.4%), and diazinon (14.5%). The second class of pesticide most commonly applied was pyrethroids, which had been identified in the following proportion: esfenvalerate (24%), lamdacyalothrin (17.3%), permethrin (16.5%), cypermethrin (12.8%), and tau-fluvalinate (10.5%). Of the carbamates, approximately 80% were methomyl or carbaryl, and of the organochlorines, 60% of all applications were dienochlor. With these findings, it was found that the proximity of OPs, at some point during pregnancy, was associated with an increased risk of 60% for ASD, greater for exposures in the third trimester (OR=2.0), and application of CPF in the second trimester (OR=3.3). Children of mothers who live close to pyrethroid insecticide application shortly before conception or during the third trimester had a higher risk of ASD and developmental delay (DD), with ORs ranging from 1.7 to 2.3. The risk of DD increased in the vicinity of carbamate pesticide applications, but no specific vulnerable period was identified. Among those exposed, only one-third were exposed to a single compound over the course of the pregnancy.

According to the study by Rauh et al.^33^ CPFs were likely to cause clinically significant tremor in all four dichotomous measures of tremor in boys when analyzed using the chi-square test (dominant arm p=0.008; non-dominant arm p=0.045; any arm p=0.022; and in both arms p=0.015).

In addition to neurodevelopmental problems, CPFs were associated with decreased motor function at 9 months of age, as demonstrated by Silver et al.,^34^ where prenatal exposure, mainly by female children, to OP insecticides was more sensitive to negative effects on motor function.

In animals models, De Felice et al. observed that strains of mice with idiopathic autism with prenatal exposure to CPF insecticide indicate significantly delayed motor maturation, the persistence of immature patterns such as pivoting at the expense of coordinated locomotion and a tendency toward enhanced ultrasound vocalization,^35^ and major oxidative stress processes.^36^

Berg et al.^37^ found that rats’ CPF exposure reflects in behavior and in some possible neuroanatomical differences, especially in those that are highly relevant in neurodevelopmental disorders, including autism spectrum disorder. The effects that were observed in both sexes at multiple time points and that did not inhibit acetylcholinesterase activity in the brain or blood suggest that current regulations regarding safe levels of CPF need to be reconsidered.

Von Ehrenstein et al.^23^ noted the risk of developing ASD associated with prenatal exposure to glyphosate (OR=1.16), CPF (OR=1.13), diazinon (OR=1.11), malathion (OR=1.11), avermectin (OR=1.12), and permethrin (OR=1.10). In addition, the risk of developing ASD with comorbid intellectual disability (ID) was associated with prenatal exposure to glyphosate (OR=1.33), CPF (OR=1.27), diazinon (OR=1.41), permethrin (OR=1.46), methyl bromide (OR=1.33), and myclobutanil (OR=1.32). In case of exposure in the first year of life, the chance of developing ASD with IDs increased by up to 50% for some pesticide substances, such as glyphosate and diazinon.

Pu et al.^38^ detected ASD-like behavioral abnormalities in rat pups after maternal exposure to high levels of glyphosate and abnormal composition of the intestinal microbiota and short-chain fatty acids.

Zhang et al.,^39^ in his meta-analysis, did not verify the relationship between glyphosate and ASD, but reported evidence from studies on humans, animals, and mechanics of a convincing link between exposure to glyphosate-based herbicides (GBHs) and increased meta-relative risk (meta-RR) of non-Hodgkin lymphoma (NHL) increased by 41% (meta-RR=1.41, 95%CI 1.13–1.75).

As the main results, the study by Cheslack-Postava et al.^13^ observed that in all the participants’ prenatal serum samples, POPs, except for DDE and HCB, were present at levels above the detection limit. Total PCBs at or above the 90th percentile were associated with an OR=1.91 for autism, suggesting that qualitatively higher levels of total PCBs may be associated with ASD risk.

The effects of PCBs on the results of neurodevelopment are consistent across different studies, as evidenced by the systematic review conducted by Berghuis and Roze.^40^ Most studies reported inverse associations with neurodevelopmental outcomes, which is caused due to exposure not only in the perinatal period but also in adolescence.

According to the systematic review by Panesar et al.^16^ from 12 articles analyzed, 8 of them found associations of developmental PCB exposure with deficits in at least one measure of cognition. It is stated that PCBs modulate the signaling pathways involved not only in the main symptoms of ASD but also in its associated comorbidities. However, it is important to note that not all PCB congeners act in the same way in the development of toxicity, as there is a difference between them and their metabolites.

The work conducted by Lyall and collaborators^20^ assessed whether prenatal exposure to PCBs and organochlorine pesticides (OCPs) was associated with the development of ASD and ID without autism in the offspring. As a result, in the ASD group, the levels of PCBs and trans-nanochlorines were higher than in the CG (control group). In addition, higher blood levels of PCBs 138/158, 153, 170, and 180 (AOR>1.5) have been associated with the development of ASD.

In addition to neurotoxic properties,^41^ persistent organic pollutants (POPs) are also associated with attention-deficit hyperactivity disorder (ADHD) form.^42^ The study realized by Rosenquist et al.^43^ investigated the association between prenatal and childhood exposures to PCB-153 and p,p’-DDE and behavior in children aged between 5 and 9 years. A high OR score was observed for hyperactivity, with results for prenatal (OR=1.24) and postnatal (OR=1.08) PCB-153 and for prenatal (OR=1.43) and postnatal (OR=1.27) p,p’-DDE. Therefore, early exposure to p,p’-DDE and PCB-153 was associated with a higher prevalence of abnormal hyperactivity scores in the study population.

The study conducted by Brown et al.^19^ concluded an evidence that maternal exposure to insecticides is associated with autism among children, as the chances of autism in the offspring increased significantly when maternal levels of p,p’-DDE were in the upper 75th percentile of the control distribution (OR=1.32). The association between maternal levels of DDE greater than the 75th percentile and the ASD chances was significant between male children (OR=1.35) and among cases of individuals with IDs (OR=2.21), but there was no association between the levels of total PCBs and maternal counterparts with ASD.

Evidence that DDE levels may be associated with the cognitive development of these children was found in the study by Gaspar et al.,^44^ in which prenatal DDE levels in female children were inversely associated with IQ (p=0.01) and processing speed at the age of 7 years (verbal comprehension, p=0.04 and processing speed, p=0.01).

The findings of this review are limited by the small number of articles included. Among the selected studies, in addition to the limited sample size,^13^ lack of estimated exposure to other potential sources, such as food,^22,23^ use of chemicals,^20^ smoking,^23^ and other environmental factors,^13^ as well as the non-pairing of specific factors in each study were mentioned as limiting factors.^19,13^ We also cited the possible errors in the pesticide application database,^22^ as well as the change of address without knowledge.^21,23^

In addition to the limiting factors of the articles presented, the quality tool used in this systematic review, the Newcastle-Ottawa Scale, does not have a classification of the studies under evaluation, requiring standardization among researchers.

 In conclusion, the studies provided found high rates of association between early exposure to agricultural pesticides and autism, which are mainly related to organochlorines, OPs, carbamates, and pyrethroids. In addition to the observation that maternal biomarkers for p,p’-DDE were increased in cases of autism and autism with IDs, divergences were found in the case of PCBs. Sample of PCBs, HCB, and DDE had detection limit above the quantification. With this, we conclude that there are studies regarding the premature exposure to agricultural pesticides and the development of ASD. However, these results should be considered with caution, due to these methodological limitations and also lack of evaluation of related genetic and environmental factors. It is hoped that the results collected by this systematic review can contribute to the development of other studies, including genetic and environmental factors. Knowing its limitations, it is necessary to continue and improve future studies for a better understanding and correlation between pesticides and autism spectrum disorder.

As stated, an urgent approach strategy is needed to reduce the use of pesticides and encourage the adoption of agroecological practices in order to ensure food and nutritional security population, as well as the reduction of risk to human and environmental health.
